# Zinc contents in foods and estimates of dietary intakes in Guangzhou, Guangdong Province, China

**DOI:** 10.3389/fnut.2024.1364033

**Published:** 2024-03-06

**Authors:** Wenqiang Mai, Fan Wang, Shuyou He, Yanmao Wen, Guanghui Yu, Lei Zhang, Hanying Dong

**Affiliations:** ^1^School of Atmospheric Sciences, Sun Yat-sen University, Zhuhai, China; ^2^Foshan Meteorological Service, Foshan, China; ^3^Guangxi Green Hope Investment Co., Ltd., Nanning, China; ^4^School of Environmental Science and Engineering, Sun Yat-sen University, Guangzhou, China; ^5^School of Resource & Environment and Safety Engineering, Hunan University of Science and Technology, Xiangtan, China; ^6^Zhongkai University of Agriculture and Engineering, Guangzhou, China

**Keywords:** zinc, Guangzhou, food composition, dietary intake, health risk

## Abstract

Zinc is one of human essential metals. In this study, 101 kinds of daily food for residents, including vegetables, aquatic food, meat, fruits, rice and cereal products, pulse food, dairy products and eggs, were collected at various agricultural markets and supermarkets in Guangzhou city, China, and their zinc contents were determined. The results showed oyster is most abundant in zinc (703.5 ± 25.6 mg/kg), followed by high-zinc milk powder (58.63 ± 0.90 mg/kg), pulse food, mutton, beef and pig liver with zinc contents above 30 mg/kg. The zinc contents of rice and cereal products, milk powder, poultry, pork, some aquatic food and eggs are also relatively high (>10 mg/kg), while vegetables and fruits have zinc contents significantly below 10 mg/kg. The daily zinc intake per person was determined by considering the zinc content of various food types and the dietary habits of specific demographic groups, resulting in 12.3 mg/day for the normal person, 11.2 mg/day for low-income individual, 12.3 mg/day for middle-income individual, 13.3 mg/day for high-income individual, 10.2 mg/day for older individual, 12.9 mg/day for factory worker, 11.5 mg/day for college student, and 8.4 mg/day for kindergarten child. The reference values of zinc intake recommended by the Chinese Nutrition Society were used to evaluate the zinc intake of Guangzhou residents, showing that the residents’ zinc intake is generally sufficient and not necessary to use zinc supplementation. Income, age and occupation could have posed influence on dietary intake of zinc.

## 1 Introduction

Zinc is an essential nutrient element for the human body, widely distributed in almost all organs or tissues, including skin, hair, blood, bone, liver, kidney, testis, epididymis, eye, prostate and others, in the form of cytosol or mitochondrial metalloproteins (1, 2). Despite constituting only 0.003% (1.4–2.3 g) of a healthy adult human body, zinc is required by more than 300 enzymes, playing an indispensable role in cellular metabolic processes such as protein synthesis, immune system function, and gene expression regulation (3). As a result, zinc deficiency can lead to a variety of disorders in the human body, such as visual impairment, myocardial damage, fetal malformation, anorexia and hypoimmunity (4, 5). In children, zinc deficiency can cause growth retardation, anorexia, decreased physical activity, intellectual development disorders and neurobehavioral abnormalities, as well as increased risks of diarrhea and respiratory infection (6–11). Conversely, excessive zinc intake can lead to acute zinc poisoning, characterized by symptoms such as nausea, vomiting, acute abdominal pain, diarrhea and fever. Chronic zinc poisoning is mainly manifested by anemia, decreased immunity, and reduced high-density lipoprotein cholesterol (12).

Since the human body can rapidly mobilize a quantity of endogenous zinc for metabolic processes, a consistent intake of sufficient zinc is essential to maintain physiological functions. The zinc content in the human body depends on the amount of zinc intake from food. Nevertheless, zinc deficiency remains a global problem. Infants and children, adolescents, pregnant women, older adults and vegetarians are groups with high incidences of zinc deficiency diseases (13). It has been reported that there is a prevalence of inadequate zinc intake in approximately 20% of the population (1, 13). The Lancet series studies focusing on undernutrition in pregnant women and children found that zinc deficiency contributes to about 4% of child mortality and disability-adjusted life-years (14). Consequently, many low- and lower-middle-income countries have made zinc inclusion mandatory in wheat flour, maize flour or rice (15).

Since 1952, the nationwide Chinese food composition tables have been continuously updated and improved. However, regional investigations of the zinc contents of local foods in China are relatively limited (16, 17), and most are restricted to reports only in Chinese language, overlooking significant differences in diet habits between provinces or population groups. Moreover, significant changes have occurred in the dietary habits of urban residents in China over time. For example, there has been an increase in the consumption of high-calorie, high-fat, low-fiber foods, resulting in changes in the intake of zinc, a topic that has been rarely discussed. In this study, a comprehensive survey was conducted by measuring the zinc contents in local foods in Guangzhou, the third largest city in China renowned for its Cantonese cuisine which highlights the freshness of ingredients and the finesse of culinary techniques. The daily zinc intake of residents was then evaluated by combining the investigation data with the dietary habits of different demographic groups in Guangzhou. The aim is to provide a holistic view into zinc contents in food and dietary intake by local populations in Guangzhou and establish a scientific foundation for promoting a balanced and nutritious diet.

## 2 Methods and material

### 2.1 Study area and sampling

Guangzhou, the provincial capital of Guangdong province, ranks among the largest cities in China, with a population of 18.81 million and covering the area of approximately 7,434 km^2^. The city experiences an annual rainfall of about 1,000 mm, with a mean annual temperature of 16°C. Guangzhou is renowned for its Cantonese cuisine, one of the eight major culinary traditions known for its authenticity, mild and delicate flavors. In this study, food samples from 101 different types were randomly collected between April and December of 2005 from the most popular 4 local wholesale markets, 7 farmers’ markets and 4 chain supermarkets in different regions of Guangzhou. For each type of vegetable, fruit, aquatic food and egg, 3 ~ 5 samples were collected and combined into one sample for further analysis. Additionally, more than 1 kg of rice and cereal products, tofu products, pulse food, meat and dairy products were collected for each sample. All fresh samples were promptly transported to laboratory and stored in the refrigerator before analysis.

### 2.2 Sample treatment and zinc analysis

The samples of vegetable, fruit, meat and aquatic food were rinsed 3 to 5 times with tap water to remove flying dust, soil or impurities that could interfere with the determination of the samples. Subsequently, they were rinsed 2 to 3 times with distilled water, followed by 3 rinses with Milli-Q ultrapure water (18.2 MΩ·cm^−1^). Finally, the samples were wiped dry with clean dry gauze or coarse filter paper.

Rice, flour, tofu products and similar items were thoroughly mixed. Inedible parts of fruits and vegetables were initially removed according to citizens’ habits. Small fruits and vegetables were uniformly mixed and sampled using the quartering method, while large fruits and vegetables were collected from multiple pieces and pooled. Leafy vegetables were sampled from different parts. Meat and aquatic food were deboned and sampled from various parts. Eggs were shell-free and stirred well. All samples were crushed and homogenized. Liquid milk and yogurt were well-shaken before weighing.

The sample digestion followed the procedure outlined in GB/T5009.14-1996. A mass of 2.000 g ~ 5.000 g of samples was weighed into a 150 mL high-type beaker. Subsequently, 15 mL concentrated nitric acid was added, and the beaker was covered with a watch glass. The mixture was left to soak overnight. The beaker was then placed on an electric heating plate and gentle heating ensured until the particles were completely dissolved. Later, 5–10 mL of concentrated nitric acid and 3–5 mL of perchloric acid were added. The solution was shaken, and gradually heated. As the heating continued, the solution gradually thickened, acquiring a brownish-red color. Care was taken to prevent ashing during this process. An additional 5–10 mL concentrated nitric acid was added. If a brownish-red ashing trend persisted, more concentrated nitric acid was added. The heating and digestion process continued until the solution became transparent and colorless. Afterwards, the solution was further evaporated until thick white smoke emitted, and pink or yellow-white residue appeared. The beaker was then removed from heat for cooling. The solution was transferred into a 25 mL volumetric flask and diluted to the scale. The resulting solution was then transferred into a plastic bottle for standby.

After digestion, the zinc content was determined using atomic absorption spectrophotometry (Hitachi Z-5000). Precision experiment, the parallel sample method, and spiking recovery experiment were employed to ensure the accuracy of determination. In each batch of samples, three reagent blanks, two standard spikes and two randomly selected parallel samples were measured for quality control. Deviations between the two parallel samples were consistently within 5%. The recovery rate of environmental standard samples (tomato leaves, 36.15 ± 3.09 mg/kg) from the National Reference Material Center of China exceeds 90%.

### 2.3 Survey on dietary habits

The daily food intake of specific groups of individuals were obtained from a comprehensive survey conducted by the Guangdong Provincial Health Department, Department of Science and Technology, and Bureau of Statistics in 2002, as provided in the Supplementary Tables S1, S2. In this survey, a 24-h recall method was employed to assess the daily dietary intake of each family member aged 2 years and older in the sampled households over three consecutive days, including meals eaten outside the home. The collected data were then standardized based on factors such as age, gender, and level of physical activity for a “normal person” and were categorized into low-income, middle-income and high-income groups. To gather information on the dietary habits of specific demographic groups, questionnaires were issued to 100 older individuals in five nursing homes, 200 college students in five boarding universities/colleges, and 120 factory workers in five boarding factories, respectively, over a span of three consecutive days. For children, their dietary habits were determined by measuring both raw and cooked food consumed during meals and recording the number of children of different ages at 111 kindergartens (totaling 19,231 children) over a span of five consecutive days.

### 2.4 Statistical analysis and calculation

The food classification was according to China Food Composition Table in 2002. The zinc content of each type of food is the average value of all the tested samples. The significance of the difference between food varieties was analyzed by SPSS 12.0 average *t*-test method, and the statistical significance level is *p* < 0.05. The daily intake of zinc per person (I) is calculated as the sum of the product of the zinc content and the amount of daily intake of each type of food:


(1) I=A1×B1+A2×B2+A3×B3+……+An×Bn

where A_n_ represents the weighted content of zinc in the n^th^ food type; B_n_ represents the daily intake of the n^th^ food type per person (see Supplementary Tables S1, S2). The weighted zinc contents of various food types (A values) are calculated based on the proportion of certain foods in the diet of Guangzhou residents according to the survey of dietary habits and the daily sales proportions of various foods in the Guangzhou market (Supplementary Table S3).

## 3 Results

### 3.1 Zinc contents in vegetables

There are variations in zinc contents among different vegetable varieties as listed in Table 1. Leafy vegetables exhibit a zinc content ranging from 5.68 ± 0.43 to 1.23 ± 0.53 mg/kg. Elevated levels are detected in salted potherb mustard, garden sass, broccoli and cabbage, all exceeding 4.50 mg/kg, while lower zinc levels are found in purple amaranthus, cauliflower, bitter cress and celery, below 2.00 mg/kg. Edible fungi have a zinc content range of 5.88 ± 0.30 to 0.84 ± 0.09 mg/kg, with straw mushroom recording the highest content and black fungus the lowest. Kelp, one of the representative marine plants, has a high zinc content of 6.34 ± 0.88 mg/kg. Among gourd vegetables, the zinc content spans from 1.80 ± 1.33 to 0.62 ± 0.05 mg/kg, with wax gourd, Yunnan small melon, cucumber, and sponge gourd showcasing significantly higher levels above 1.20 mg/kg than others. In the solanaceous vegetable group, bell pepper has the highest zinc content at 1.46 ± 0.45 mg/kg, which is statistically similar to chilli (1.30 ± 0.13 mg/kg) but significantly higher than eggplants (1.01 ± 0.15 mg/kg) and tomatoes (0.46 ± 0.23 mg/kg). The zinc content range of legume vegetables is 3.85 ± 0.15 to 2.46 ± 0.47 mg/kg, with broad beans and long beans exhibiting significantly higher zinc contents than hyacinth beans and green beans. Among the bulb vegetables, chive has the highest zinc content at 2.47 ± 1.11 mg/kg, whereas onion shows the lowest at only 1.27 ± 0.26 mg/kg, but the differences among chives, garlic, and scallions are not significant. Regarding root/stem vegetables, the zinc content varies from 2.28 ± 0.41 to 0.64 ± 0.05 mg/kg; taro and potatoes have the highest zinc contents, while asparagus lettuce has the lowest, significantly different from other varieties.

**Table 1:** Zinc contents in the raw vegetables (unit: mg/kg, fresh weight).

Food description	Mean ± stdev (*n*)	Range	Food description	Mean ± stdev (*n*)	Range
*Leaf vegetables*			*Gourd vegetables*		
Salted potherb mustard	5.68 ± 0.43(4)	5.22–6.25	Wax gourd	1.80 ± 1.33(5)	0.61–3.62
Garden sass	4.65 ± 0.30(6)	4.21–419	Yunnan small melon	1.40 ± 0.56(6)	0.91–2.28
Broccoli	4.55 ± 1.60(8)	2.84–6.69	Cucumber	1.38 ± 0.43(6)	0.94–2.10
Cabbage	4.50 ± 0.37(5)	4.34–5.23	Sponge gourd	1.28 ± 0.36(5)	0.74–1.65
Pea seedling	3.22 ± 0.52(6)	2.85–4.10	Chayote	1.04 ± 0.27(7)	0.77–1.35
Spinach	3.06 ± 0.65(8)	2.52–4.15	Hairy gourd	0.82 ± 0.25(7)	0.60–1.21
Chinese kale	3.02 ± 0.75(7)	1.70–2.62	Bitter gourd	0.80 ± 0.19(7)	0.63–1.12
Chinese parsley	2.87 ± 0.91(7)	1.88–4.04	Pumpkin	0.62 ± 0.05(5)	0.61–0.73
Bean sprouts	2.85 ± 0.98(6)	1.73–4.00	*Solanaceous vegetables*		
Leaf mustard	2.80 ± 0.43(7)	2.44–3.53	Bell pepper	1.46 ± 0.45(6)	0.92–2.02
Water spinach	2.60 ± 0.26(6)	2.44–3.03	Chilli	1.30 ± 0.13(5)	1.15–1.48
Lettuce	2.57 ± 0.72(8)	1.66–3.80	Eggplant	1.01 ± 0.15(6)	0.85–1.24
Chinese cabbage	2.56 ± 0.64(4)	1.64–3.57	Tomato	0.46 ± 0.23(10)	0.22–0.77
Chinese flowering cabbage	2.55 ± 0.39(5)	2.24–3.32	*Legume vegetables*		
Shanghai bok choy	2.28 ± 0.40(6)	1.70–2.64	Broad bean	3.85 ± 0.15(9)	3.74–4.02
Bok choy	2.22 ± 0.74(7)	1.65–3.65	Long bean	3.20 ± 0.23(8)	3.01–3.54
Water cress	2.18 ± 1.01(5)	1.40–3.66	Hyacinth bean	2.64 ± 0.37(7)	2.33–3.24
Indian lettuce	2.04 ± 0.74(8)	1.60–3.72	Green bean	2.46 ± 0.47(7)	2.02–3.01
Purple amaranthus	1.96 ± 0.47(6)	1.55–2.66	*Bulb vegetables*		
Cauliflower	1.83 ± 0.58(8)	1.10–2.64	Chive	2.47 ± 1.11(8)	1.68–4.83
Bitter cress	1.24 ± 0.27(5)	0.80–1.52	Garlic	2.06 ± 0.76(7)	1.25–2.94
Celery	1.23 ± 0.53(6)	0.72–2.10	Scallion	1.50 ± 0.27(6)	1.35–1.97
*Edible fungi*			Onion	1.27 ± 0.26(8)	0.95–1.61
Straw mushroom	5.88 ± 0.30(5)	5.31–6.08	*Root/stem vegetables*		
Dried mushroom	4.23 ± 1.50(7)	2.28–5.98	Taro	2.28 ± 0.41(10)	1.81–2.86
Needle mushroom	3.22 ± 0.40(5)	2.84–3.75	Potato	2.00 ± 0.47(10)	1.42–2.64
Oyster Mushroom	3.08 ± 0.72(6)	2.33–3.79	Lotus root	1.42 ± 0.52(7)	0.81–2.13
black fungus	0.84 ± 0.09(6)	0.72–0.94	Carrot	1.23 ± 0.45(10)	0.92–2.04
*Marine plants*			Radish	1.08 ± 0.20(10)	0.96–1.40
Kelp	6.34 ± 0.88(6)	5.20–7.34	Asparagus lettuce	0.64 ± 0.05(6)	0.64–0.78

The zinc levels among the eight categories of vegetables show significant variations, with the following order: marine plants 6.34 ± 0.88 mg/kg > edible fungi 3.41 ± 1.84 mg/kg > legume vegetables 3.07 ± 0.63 mg/kg > leafy vegetables 2.79 ± 1.09 mg/kg > bulb vegetables 1.95 ± 0.54 mg/kg > root/stem vegetables 1.45 ± 0.61 mg/kg > gourd vegetables 1.13 ± 0.39 mg/kg > solanaceous vegetables 1.02 ± 0.44 mg/kg.

### 3.2 Zinc contents in aquatic food

The contents of zinc in the raw aquatic food are listed in Table 2. The range of zinc content in freshwater fish is 25.47 ± 0.99 to 6.49 ± 0.77 mg/kg, with loach having the highest zinc content, followed by mud eels at 17.52 ± 0.72 mg/kg and crucian carp, dace, tilapia, and grass carp all around 10 mg/kg, while the lowest is found in catfish and perch (7.04 ± 1.04 mg/kg). The zinc content in marine fish spans from 16.05 ± 1.36 to 4.75 ± 0.34 mg/kg, with rabbit fish having the highest, followed by silver sillago at 13.24 ± 0.92 mg/kg, while hairtail fish, bummalo, bream and herring register the lowest. Among mollusks, squid has the highest zinc content at 18.75 ± 0.92 mg/kg, significantly outstripping jellyfish, octopus and cuttlefish at 14.00 ± 0.16 mg/kg, 15.30 ± 0.67 mg/kg and 15.85 ± 2.19 mg/kg, respectively. As for crustaceans, the zinc contents in two oyster samples are approximately 30 times higher than in crabs (24.83 ± 2.75 mg/kg), clams (23.72 ± 1.21 mg/kg), and shrimps (13.07 ± 1.28 mg/kg).

**Table 2:** Zinc contents in the raw aquatic food (unit: mg/kg, fresh weight).

Food description	Mean ± stdev (*n*)	Range	Food description	Mean ± stdev (*n*)	Range
*Freshwater fish*			*Marine fish*		
Loach	25.47 ± 0.99(6)	24.59–26.43	Rabbit fish	16.05 ± 1.36(4)	14.65–17.86
Mud eel	17.52 ± 0.72(5)	16.17–18.02	Silver sillago	13.24 ± 0.92(8)	12.46–14.35
Carp	14.39 ± 1.11(8)	13.37–15.87	Boston mackerel	10.56 ± 2.68(7)	7.59–13.05
Crucian carp	11.73 ± 1.68(6)	10.30–14.14	Saury	10.01 ± 1.13(6)	8.78–11.45
Dace	11.67 ± 0.65(4)	10.95–12.72	Golden pomfret	7.70 ± 0.66(5)	6.87–8.68
Tilapia	10.10 ± 1.71(8)	8.15–12.65	Yellow croaker	7.49 ± 0.52(8)	7.09–8.25
Grass carp	8.92 ± 2.47(8)	6.11–12.72	Hairtail fish	6.32 ± 0.32(8)	5.89–6.64
Perch	7.04 ± 1.04(7)	5.75–8.44	Bummalo	5.86 ± 0.57(4)	5.06–6.54
Catfish	6.49 ± 0.77(4)	5.45–7.62	Bream	5.53 ± 0.61(5)	4.54–6.05
			Herring	4.75 ± 0.34(6)	4.48–5.23
*Mollusks*			*Crustaceans*		
Squid	18.75 ± 0.92(5)	17.43–19.60	Crab	24.83 ± 2.75(11)	20.87–28.98
Octopus	15.30 ± 0.67(13)	12.56–18.05	Oyster	703.5 ± 25.6(2)	685.4–721.6
Cuttle fish	15.85 ± 2.19(8)	13.32–19.59	Clam	23.72 ± 1.21(12)	12.94–38.52
Jelly fish	14.00 ± 0.16(7)	12.00–15.60	Shrimp	13.07 ± 1.28(12)	9.92–15.00

No significant differences in zinc content were observed between mollusk and freshwater fish categories, but significant differences exist between other aquatic food categories. The zinc contents across the four different aquatic food categories follow the order: crustaceans (excluding oyster) 19.91 ± 9.65 mg/kg > mollusks 13.78 ± 2.19 mg/kg ≈ freshwater fish 11.44 ± 5.73 mg/kg > marine fish 8.60 ± 3.68 mg/kg. Oyster exhibits the highest zinc contents among all the studied food types.

### 3.3 Zinc contents in meat

The contents of zinc in the raw meat are listed in Table 3. In terms of red meat, beef and mutton exhibit higher zinc contents with no notable difference between them, measuring 33.57 ± 4.81 mg/kg and 31.55 ± 4.05 mg/kg, respectively, significantly higher than pork (19.71 ± 4.25 mg/kg). The descending order of zinc content in poultry is quail (24.15 ± 1.51 mg/kg) > duck (19.15 ± 3.40 mg/kg) > goose (15.14 ± 1.28 mg/kg) > chicken (12.76 ± 1.97 mg/kg), with significant differences among the four types. In pork viscera, pig liver leads with a zinc content of 34.82 ± 2.30 mg/kg, followed by pig kidney (25.96 ± 2.46 mg/kg) and pig heart (18.08 ± 0.79 mg/kg), all displaying significant differences in zinc content.

**Table 3:** Zinc contents in the raw meat (unit: mg/kg, fresh weight).

Food description	Mean ± stdev (*n*)	Range
*Red meat*		
Beef	33.57 ± 4.81(7)	26.87–39.37
Mutton	31.55 ± 4.05(8)	26.74–36.94
Pork	19.71 ± 4.25(13)	15.20–27.39
*Poultry*		
Quail	24.15 ± 1.51(4)	23.21–26.66
Duck	19.15 ± 3.40(5)	15.10–21.15
Goose	15.14 ± 1.28(6)	14.28–16.24
Chicken	12.76 ± 1.97(7)	10.29–15.39
*Pork viscera*		
Pig liver	34.82 ± 2.30(8)	31.76–37.53
Pig kidney	25.96 ± 2.46(5)	23.45–26.86
Pig heart	18.08 ± 0.79(4)	17.82–19.06

The overall zinc content varies significantly among three different categories of meat, following the order: red meat 26.40 ± 7.48 mg/kg ≈ pork viscera 26.40 ± 8.37 mg/kg > poultry 17.10 ± 4.98 mg/kg. Among all meats, pig liver has the highest zinc content, followed by mutton, beef, and quail, with chicken having the lowest zinc content.

### 3.4 Zinc contents in fruits

The contents of zinc in fruits are listed in Table 4. Among melons, papaya (5.52 ± 1.08 mg/kg) and muskmelon (5.06 ± 1.31 mg/kg) have significantly higher zinc contents compared to watermelon (3.02 ± 1.21 mg/kg) and honeydew melon (2.34 ± 0.09 mg/kg). In the realm of tropical and subtropical fruits, starfruit (7.26 ± 1.94 mg/kg), pitaya (6.36 ± 0.35 mg/kg) and banana (5.28 ± 0.92 mg/kg) have significantly higher zinc contents than guava (4.16 ± 0.72 mg/kg). Turning to stone fruits, jujube leads with a zinc content at 4.02 ± 0.33 mg/kg, followed by black plum at 2.28 ± 0.43 mg/kg, while peach has the lowest content at 1.72 ± 0.13 mg/kg. Among the citrus fruits, no significant difference exists in zinc content among orange, grapefruit, and tangerine, with levels around 2.30 mg/kg. Within berries, kiwi fruit stands out with a significantly higher zinc content at 2.06 ± 0.38 mg/kg, than persimmon at 1.59 ± 0.22 mg/kg, and grapes at 1.22 ± 0.17 mg/kg. Pome fruits reveal a significant difference, with pear having a significantly higher zinc content at 1.21 ± 0.20 mg/kg than apple at only 0.86 mg/kg.

**Table 4:** Zinc contents in fruits (unit: mg/kg, fresh weight).

Food description	Mean ± stdev (*n*)	Range
*Melons*		
Papaya	5.52 ± 1.08(6)	4.54–7.06
Muskmelon	5.06 ± 1.31(5)	3.50–6.04
Watermelon	3.02 ± 1.21(8)	1.76–4.98
Honeydew melon	2.34 ± 0.09(6)	2.24–2.50
*Tropical and subtropical fruits*		
Starfruit	7.26 ± 1.94(8)	4.52–9.10
Pitaya	6.36 ± 0.35(7)	5.96–6.80
Banana	5.28 ± 0.92(8)	3.92–6.38
Guava	4.16 ± 0.72(6)	3.52–5.36
*Stone fruits*		
Jujube fruit	4.02 ± 0.33(13)	3.02–4.90
Black plum	2.28 ± 0.31(4)	1.96–2.68
Peach	1.72 ± 0.13(6)	1.52–1.88
*Citrus fruits*		
Orange	2.42 ± 0.50(8)	1.76–3.04
Pomelo	2.32 ± 0.43(9)	1.92–3.02
Tangerine	2.30 ± 0.17(6)	2.02–2.50
*Berries*		
Kiwi fruit	2.06 ± 0.38(4)	1.78–2.46
Persimmon	1.59 ± 0.22(6)	1.28–1.86
Grape	1.22 ± 0.17(20)	0.76–1.84
*Pome fruits*		
Pear	1.21 ± 0.20(25)	0.78–1.90
Apple	0.86 ± 0.10(7)	0.70–12.20

The zinc content of each fruit category, from highest to lowest, is as follows: tropical and subtropical fruits 5.83 ± 1.34 mg/kg > melon fruits 3.91 ± 1.54 mg/kg > stone fruits 3.02 ± 1.29 mg/kg > citrus fruits 2.34 ± 0.06 mg/kg > berries 1.52 ± 0.44 mg/kg ≈ pome fruits 1.16 ± 0.36 mg/kg. Starfruit emerges as the fruit with the highest zinc content at 7.60 mg/kg, followed by pitaya at 6.48 mg/kg, while apples display the lowest zinc content at only 0.86 mg/kg.

### 3.5 Zinc contents in other food groups

Significant variations in zinc content are evident across other food groups, as detailed in Table 5. In terms of rice and cereal products, rice has the highest zinc content at 17.04 ± 1.13 mg/kg, followed by noodles at 10.90 ± 3.21 mg/kg, with the lowest levels found in flour at 10.52 ± 2.75 mg/kg. Among pulse food, peanut leads with the highest zinc content at 39.56 ± 2.08 mg/kg, followed closely by soybean at 39.13 ± 3.64 mg/kg, and mung bean at 36.33 ± 4.74 mg/kg. Notably, zinc contents in tofu (9.51 ± 2.61 mg/kg) and bean curd sheet (10.80 ± 14.90 mg/kg) are considerably lower. Within dairy products, milk powder exhibits a zinc content of 27.80 ± 1.55 mg/kg, while high-zinc milk powder surpasses that with a zinc content of 58.63 ± 0.90 mg/kg, twice the amount found in regular milk powder. The zinc content in whole milk is 3.82 ± 0.46 mg/kg, and yogurt follows with 2.31 ± 0.45 mg/kg. In the egg category, zinc content ranges from 17.31 ± 1.46 mg/kg to 9.98 ± 2.35 mg/kg.

**Table 5:** Zinc contents in other food groups (unit: mg/kg, fresh weight).

Food description	Mean ± stdev (*n*)	Range
*Rice and cereal products*		
Rice	17.04 ± 1.13(37)	13.25–21.57
Noodle	10.90 ± 3.21(11)	8.46–21.70
Flour	10.52 ± 2.75(11)	8.60–13.96
*Pulse food*		
Soybean	39.13 ± 3.64(6)	34.45–44.04
Mung bean	36.33 ± 4.74(7)	30.56–42.90
Red bean	34.19 ± 3.43(7)	31.25–39.12
Black-eye bean	34.05 ± 0.42(6)	32.14–34.53
Peanut	39.56 ± 2.08(7)	36.93–40.26
*Tofu products*		
Tofu	9.51 ± 2.61(6)	7.58–13.52
Bean curd sheet	10.81 ± 14.92(7)	17.58–50.71
*Dairy products*		
Liquid milk	3.82 ± 0.46(9)	3.03–4.13
Yogurt	2.31 ± 0.45(5)	1.68–2.81
Milk powder	27.80 ± 1.55(5)	26.32–29.81
High-zinc milk powder	58.63 ± 0.90(6)	56.27–59.72
*Eggs*		
Duck egg	17.31 ± 1.46(5)	15.43–18.92
Chicken egg	12.33 ± 1.20(11)	8.28–16.65
Preserved egg	9.98 ± 2.35(6)	8.00–12.97
Salted egg	14.39 ± 3.95(7)	10.35–19.5

## 4 Discussion

### 4.1 Comparison in zinc contents with other studies

In general, animal-based foods have higher zinc contents compared to plant-based foods. Oyster takes the lead with the highest zinc content at 703.5 ± 25.6 mg/kg, followed by high-zinc milk powder at 58.63 ± 0.90 mg/kg. Among animal-based foods, the hierarchy in zinc content across different food groups is: meat > eggs > aquatic food (excluding oyster) > dairy products (excluding high-zinc milk powder). Among plant-based foods, pulse food has the highest zinc contents, surpassing 30 mg/kg, followed closely by rice and cereal products. In contrast, fruits and vegetables tend to have the lowest zinc contents, generally falling below 10 mg/kg. Good sources of zinc in the diet include animal-based foods like oyster, beef and mutton, as well as plant-based foods like soybean, mung bean, red bean, black-eye bean and peanut.

These findings are consistent with previous studies indicating that fish, viscera, mollusk, crustacean, and meat serve as excellent animal-based sources of zinc, while pulse foods emerge as noteworthy plant-based contributors to zinc intake (18). The pattern and range of zinc contents of various food groups in Guangzhou aligns with those reported in European, Asian, African, and American countries [e.g., (19–24)] and other regions in China [e.g., (16, 17, 25, 26)]. However, the average contents of specific food groups could differ by up to one order of magnitude between studies, suggesting large regional varieties in food origins. Also, oysters in Guangzhou market with zinc contents of 703.5 ± 25.6 mg/kg is considerably higher than 23–90 mg/kg for oysters living in non-contaminated locations but slightly lower than 1,200 mg/kg for oysters in highly-contaminated areas, suggesting their origins from relatively moderately-polluted areas (27).

### 4.2 Estimated dietary intakes of zinc by Guangzhou residents

The weighted zinc contents of various food types [A values of Eq. (1)] as calculated based on the proportions of certain foods in the diet of Guangzhou residents according to the survey of dietary habits and the daily sales proportions of various foods in the Guangzhou market (Supplementary Table S3) are as follows:

- Rice 17.04 mg/kg
- Flour 10.52 mg/kg
- Vegetables 2.18 mg/kg (sales proportions in the market: 53% leaf vegetable, 1% edible fungi, 15% gourd vegetable, 12% solanaceous vegetable, 6% bean vegetable, 2% bulb vegetable, 11% root/stem vegetable)
- Fruits 2.75 mg/kg (sales proportions in the market: 70% apple, banana, pear, citrus fruit or watermelon, 30% starfruit, papaya, persimmon, or black plum)
- Pork 19.71 mg/kg
- Beef and mutton 32.56 mg/kg (sales proportions in the market: 85% beef, 15% mutton)
- Pork viscera 26.4 mg/kg
- Poultry 15.02 mg/kg (sales proportions in the market: 42% goose, 39% chicken, 18% duck, 1% quail)
- Dairy product 2.89 mg/kg (sales proportions in the market: 50% liquid milk, 50% yogurt)
- Eggs 15.21 mg/kg (sales proportions in the market: 40% fresh chicken egg, 40% fresh duck egg, 10% preserved egg, 10% salted egg)
- Aquatic food 12.08 mg/kg [sales proportions in the market: 60% freshwater fish, 20% marine fish, 8% mollusk and 12% crustacea (excluding oysters)]
- Pulse food 36.81 mg/kg

It’s important to note that for some certain food types, were not subject to testing in the study. Instead, their values were referenced from the 2002 National Representative Values of the Chinese Food Composition Table and denoted by an asterisk (*), as shown below.

^*^Other grains 18.52 mg/kg;
^*^Potato-like food 2.20 mg/kg
^*^Soybean products 16.70 mg/kg
^*^Nuts 22.7 mg/kg
^*^Vegetable oil 3.02 mg/kg
^*^Animal fat 8.05 mg/kg
^*^Pastries and snacks 10.21 mg/kg
^*^Sugar and starch 0.72 mg/kg
^*^Salt 2.41 mg/kg
^*^Sauces 2.54 mg/kg
^*^Alcohol 11.76 mg/kg

**Figure 1:**
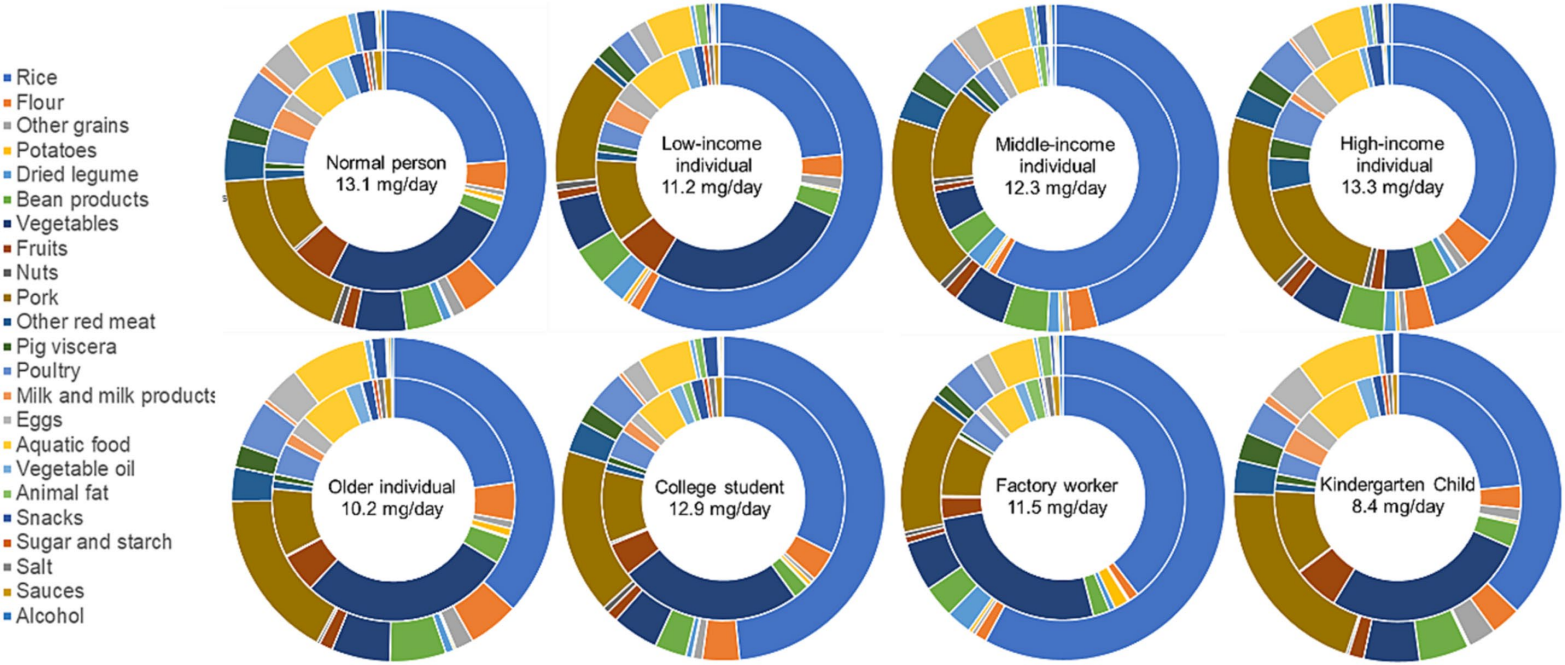
The percentages of intake weight (inner cycle) and zinc intake (outer cycle) from different food types for different demographic populations.

The daily food intake of specific groups of individuals [B values of Eq. (1)] were as shown in Supplementary Tables S1, S2. This information serves as a basis for Eq. (1) to calculate the daily zinc intake from different food sources in this study. The data depicted in Figure 1 reveals that the daily dietary zinc intake for a normal person in Guangzhou stands at 13.1 mg/day. The primary source contributing to the dietary zinc intake is rice, making up 37.9%, followed by pork at 17.4%. Vegetables and fruits account for 6.7% of the total zinc intake, whereas animal-based foods like aquatic products, meat, dairy, and eggs collectively contribute to 34.2% of the zinc intake.

In terms of varying income levels, the daily dietary zinc intake for individuals of low, middle, and high income is 11.2 mg/day, 12.3 mg/day, and 13.3 mg/day, respectively. This reveals a pattern where zinc intake increases proportionally with income. Specifically, the percentage of zinc derived from rice declines as income rises, representing 58.0, 45.8, and 35.4% sequentially. Conversely, the consumption proportion of animal-based foods escalates, accounting for 23.7, 34.4, and 41.8%, respectively. Interestingly, the ratio of zinc obtained from vegetables and fruits in relation to the total dietary zinc intake displays minimal variation across different income groups.

The daily dietary zinc intake for older individual is 10.2 mg/day. The highest percentage of dietary zinc intake is again attributed to rice, comprising 36.7%, albeit lower than the proportion observed for the normal person. Pork comes next at 17.0%. Vegetables and fruits contribute to 7.2% of the zinc intake. The consumption of animal-based foods makes a more substantial contribution, accounting for 39.2% of the dietary zinc intake, higher than the corresponding proportion of 34.4% observed for a normal person.

The daily dietary intake of zinc for college student is 12.9 mg/day. The highest percentage of dietary zinc intake is from rice, reaching 48.4%, significantly higher than the proportion for the normal person. Pork comes second at 16.5%. Vegetables and fruits contribute to 5.7% of the zinc intake. The consumption of animal-based foods contributes to 33.4% of the dietary zinc intake, not significantly different from the proportion for a normal person (34.4%).

The dietary intake of zinc for factory worker is 11.5 mg/day. The highest percentage of dietary intake of zinc is from rice, reaching 57.9%, significantly higher than the proportion for a normal person. Pork comes second at 13.7%. Vegetables and fruits contribute to 5.6% of the zinc intake. The consumption of animal-based foods contributes to 25.5% of the dietary zinc intake, significantly lower than the proportion for a normal person or the proportion from rice. Therefore, it is evident that a significant portion of dietary zinc for factory workers in Guangzhou comes from rice consumption.

The dietary zinc intake for kindergarten child aged 2 to 5 is 8.4 mg/day. The highest percentage of dietary zinc intake is associated with rice, making up 37.1%, close to the 37.9% observed for a normal person. Following rice, pork is the second-highest contributor at 20.2%, representing the highest proportion among all population groups. Vegetables and fruits contribute to 7.0% of the zinc intake. Meanwhile, animal-based foods contribute to 42.7% of the dietary zinc intake for children, notably higher than both the proportion observed for a normal person (34.4%). Therefore, for children aged 2 to 5, the majority of their dietary zinc intake comes from rice and animal-based foods. These two sources collectively account for 79.8% of the dietary zinc intake.

### 4.3 Potential health risk implications

The reference dietary intake (RI) for zinc for different demographic populations were adopted from the Chinese Nutrition Society in 2013 as: 10.0 mg/L for adults and 5.5 mg/L for children, while the “tolerable” zinc intake levels are 40 mg/L for adults and 12 mg/day for children. It can be observed that the zinc intake for all demographic populations in Guangzhou is relatively high, but not exceeding the “tolerable” intake levels. This indicates that residents in Guangzhou do not need zinc supplementation or consume foods fortified with zinc, and there would not be high risks of health issues associated with excessive zinc intake.

The dietary zinc intake of 13.1 mg/day for the normal person is relatively higher than that of 7.47 mg/day in Shenzhen (the nearby big city) obtained from the 2011 total diet investigation (17), but similar to that of 14.1 mg/day in Shenzhen from the 2008 total diet investigation (28). The changes in dietary zinc intake could mainly be attributed to the differences in the zinc content in rice between studies. In this study, rice has a zinc content that is three times higher than what was reported in the Shenzhen 2011 investigation, thus playing a major role in contributing to dietary zinc intake. Generally, the dietary zinc intake fell within the same ranges of global studies (29, 30).

These observations underscore the importance of considering specific dietary habits of different demographic groups with varying age, income and occupations, which can significantly influence the sources and proportions of nutrient intake. Rice consistently plays a significant role in contributing to zinc intake, with noteworthy differences among normal person, older individual, and college student. The prominence of animal-based foods in the diet also seems to vary, showcasing the diverse dietary habits among these groups. Especially, understanding the distribution of zinc sources in the diet of children is crucial for designing nutrition plans and interventions that cater to their specific needs during this critical developmental stage.

## 5 Conclusion

The zinc content in the diet of residents in Guangzhou varies significantly among different food categories. Animal-based food has a higher zinc content compared to plant-based foods. Among animal-based foods, the zinc contents are highest in oyster and high-zinc milk powder, followed by meat and eggs, fish and dairy products. In terms of plant-based foods, pulse food like soybean, peanut, and mung bean have higher zinc contents, followed by rice, while fruits and vegetables have the lowest zinc contents.

The zinc intake of residents in Guangzhou is associated with income levels and population groups. The dietary zinc intake for various population groups in Guangzhou, including the normal person, low-income individuals, middle-income individuals, high-income individuals, older individual, college student, factory worker, and kindergarten child, is 12.3 mg/day, 11.2 mg/day, 12.3 mg/day, 13.3 mg/day, 10.2 mg/day, 12.9 mg/day, 11.5 mg/day, and 8.4 mg/day, respectively. In the diets of these various population groups, rice represents the largest proportion, and animal-based foods also make up a significant portion. For different income groups, as income increases, zinc intake also increases. Zinc intake is generally high among various population groups in Guangzhou, with adequate intake levels.

## Data availability statement

The original contributions presented in the study are included in the article/Supplementary materials, further inquiries can be directed to the corresponding author.

## Author contributions

WM: Data curation, Formal analysis, Investigation, Writing – original draft, Writing – review & editing. FW: Supervision, Writing – original draft, Writing – review & editing. SH: Data curation, Investigation, Methodology, Writing – review & editing. YW: Conceptualization, Funding acquisition, Project administration, Resources, Supervision, Writing – review & editing. GY: Investigation, Writing – review & editing. LZ: Investigation, Writing – review & editing. HD: Conceptualization, Data curation, Investigation, Methodology, Supervision, Writing – original draft, Writing – review & editing.
