# Supplementary material for: Zinc contents in foods and estimates of dietary intakes in Guangzhou, Guangdong Province, China
Source: Front Nutr. 2024 Mar 6;11:1364033. doi: 10.3389/fnut.2024.1364033 (PMC10951084; doi:10.3389/fnut.2024.1364033)
Supplement: Supplementary file 1 [file Table_1.pdf]

Supplementary Material

Zinc contents of foods and estimates of dietary intakes in Guangzhou, Guangdong Province, China

Wenqiang Mai[1,2], Fan Wang[1], Shuyou He[3,4], Yanmao Wen[4], Guanghui Yu[5], Lei Zhang[6], Hanying Dong[1*]

[1]School of Atmospheric Sciences, Sun Yat-sen University, Zhuhai 519082, China

[2]Foshan Meteorological Service, Foshan 528010, China

[3]Guangxi Green Hope Investment Co. Ltd, Nanning 530028, China

[4]School of Environmental Science and Engineering, Sun Yat-sen University, Guangzhou 510006, China

[5]School of Resource & Environment and Safety Engineering, Hunan University of Science and Technology, Xiangtan 411201, China

[6]Zhongkai University of Agriculture and Engineering, Guangzhou 510225, China

Corresponding author: eesdhy@mail.sysu.edu.cn

Table S1 Daily intake food weight for a normal person or per individual in households of low, middle, or high-income levels

Food type	Normal person	Low-income household	Middle-income household	High-income household
Rice	290.7	380.3	331.8	276.7
Flour	48	12.1	31.6	51.1
Other grains	9.1	2.2	4.7	9.6
Potato-like food	10.2	24.2	20.6	9.5
Pulse food	3.3	7.9	4.1	4.2
Bean products	29.1	25.7	32.5	30.7
Vegetables	313.8	270.3	294	322.5
Fruits	70.1	37.4	60.3	81.6
Nuts	4.8	3.7	4.1	5.4
Pork	122.5	71.4	108.8	122.1
Beef and mutton	16.8	2.7	11.7	17.7
Viscera	10.9	6.8	10.4	13.2
Poultry	57.4	21.1	41	62.7
Dairy food	38.8	2.8	14.9	49.1
Eggs	27.6	13.6	19.7	30
Aquatic food	70.8	41.6	50.9	74.4
Vegetable oil	37.5	16.5	33.5	41.8
Animal fat	0.1	15.3	5.3	0.3
Pastries and snacks	25.2	4.5	12.5	28
Sugar and starch	7.4	3.4	7.1	7.2
Salt	8.7	10.6	10.3	9.1
Sauce	14.4	9.7	11.9	15.5
Alcohol	5.5	4.3	4.3	8.7

Table S2 Daily intake food weight of different foods for various demographic groups in Guangzhou city

Food type	Elderly resident	College student	Factory worker	Kindergarten child
Rice	219.8	365.3	389.5	181.6
Flour	49.3	45.2	13.2	23.5
Other grains	10.2	6.5	2.3	12.3
Potato food	10.4	6.5	18.9	2.6
Pulse food	2.3	2.1	7.8	0.3
Bean products	34.2	24.3	21.9	24.5
Vegetables	274.1	274.9	259	210.2
Fruits	50.3	50.3	27.9	46.3
Nuts	1.3	3.5	2.2	0.9
Pork	87.9	107.6	79.6	85.8
Beef and mutton	10.6	12.3	2.5	8.6
Viscera	8.6	9.8	5.7	8.7
Poultry	40.3	42.3	31.2	23.4
Dairy food	15.6	18.5	3	25.4
Eggs	25.6	17.5	14	22.3
Aquatic food	64.2	56.4	43.2	55.6
Vegetable oil	21.1	20.6	14.6	16.8
Animal fat	1.1	12.7	18.2	0.1
Pastries and snacks	14.2	19.6	4.1	9.8
Sugar and starch	5.6	7.2	2.8	5.3
Salt	8.6	10.3	10.4	5.3
Sauce	10.3	11.3	10.2	5.8
Alcohol	2.3	1.3	4.5	0

Table S3 Daily sales proportions of various foods in the Guangzhou market

Aquatic food		Vegetables		Eggs		Fruits		Poultry		Red meat	
**Type**	**Proportion**	**Type**	**Proportion**	**Type**	**Proportion**	**Type**	**Proportion**	**Type**	**Proportion**	**Type**	**Proportion**
Freshwater fish	60%	Leaf vegetables	53%	Chicken egg	80%	Common fruits (apple, banana, pear, orange, pomelo, tangerine, water melon, honey melon)	70%	Goose	42%	Beef	85%
Marine fish	20%	Root/stem vegetables	11%	Preserved egg	10%	Uncommon fruits (persimmon, starfruit, papaya, black plum etc.)	30%	Chicken	39%	Mutto n	15%
Mollusks	8%	Gourd vegetables	15%	Salted egg	10%			Duck	18%		
Crustaceans	12%	Solanaceous vegetables	12%					Quail	1%		
		Legume vegetables	6%								
		Bulb vegetables	2%								
		Edible fungi	1%								
